# Assessing the spatial occupation and ecological impact of human activities in Chengguan district, Lhasa city, Tibetan Plateau

**DOI:** 10.1038/s41598-024-57221-9

**Published:** 2024-03-23

**Authors:** Lin Xu, Yong Xu, Jian Duan, Yingying Wang, Hua Yang

**Affiliations:** 1 Key Laboratory of Regional Sustainable Development Modeling, Institute of Geographic Sciences and Natural Resources Research (IGSNRR), Chinese Academy of Sciences (CAS), Beijing, 100101 China; 2 College of Resources and Environment, University of Chinese Academy of Sciences, Beijing, 100049 China; 3 College of Geography and Environmental Sciences, Zhejiang Normal University, Jinhua, 321004 China; 4 Key Laboratory for Geographical Process Analysis and Simulation of Hubei Province and School of Urban and Environmental Sciences, Central China Normal University, Wuhan, 430079 China

**Keywords:** Tibetan Plateau, Human activities, Spatial occupation, Ecological impact, Carbon sequestration and oxygen release, Net primary productivity, Ecology, Urban ecology

## Abstract

In this study, the ecological impact of human activities and the space occupied by construction and arable land on the Tibetan Plateau were examined, focusing on changes in the net primary productivity (NPP) as a key indicator of ecological health. With the utilization of land use data and multiyear average NPP data from 2002 to 2020, we analyzed the effects of the conversion of zonal vegetation into construction and arable land on carbon sequestration and oxygen release in Chengguan District, Lhasa city. Our findings indicated a marked spatial difference in the NPP among different land types. Regarding the original zonal vegetation, the NPP ranged from 0.2 to 0.3 kg/m^2^. Construction land showed a decrease in the NPP, with values ranging from 0.16 to 0.26 kg/m^2^, suggesting a decrease in ecological productivity. Conversely, arable land exhibited an increase in the NPP, with average values exceeding 0.3 kg/m^2^. This increase suggested enhanced productivity, particularly in regions where the original zonal vegetation provided lower NPP values. However, this enhanced productivity may not necessarily indicate a positive ecological change. In fact, such increases could potentially disrupt the natural balance of ecosystems, leading to unforeseen ecological consequences. The original zonal vegetation, with NPP values ranging from 0.12 to 0.43 kg/m^2^, exhibited higher ecological stability and adaptability than the other land types. This wider NPP range emphasizes the inherent resilience of native vegetation, which could sustain diverse ecological functions under varying environmental conditions. These findings demonstrate the urgent need for sustainable land use management on the Tibetan Plateau. This study highlights the importance of considering the ecological impact of land use changes in regional development strategies, ensuring the preservation and enhancement in the unique and fragile plateau ecosystem.

## Introduction

Human activities constitute a core factor influencing the global ecological environment and remain a constant theme in global environmental change research. The Tibetan Plateau, as an important global ecological barrier, notably impacts the global climate and Asian water resources^1,2^. The study of human activities in this sensitive and crucial ecological environment is important and practical, representing a focal and challenging topic in geographic, ecological, and global change research^3–5^. The rapid expansion of constructed land has caused severe ecological degradation, loss of natural vegetation, disruption of surface element flows^6^, and a decrease in the carbon sequestration and oxygen release capabilities of ecosystems^7^. At present, plateau ecosystems face several environmental challenges^8,9^, including climate change, land use change, and various forms of pollution^10^. Due to its complex terrain, high altitude, and distinct climate, the Tibetan Plateau is highly sensitive to global changes^11^ and is regarded as a natural laboratory for studying climate change in China and globally^12,13^. Thus, researching the impact of human activity space occupation on the ecological environment of the plateau is important.

Recently, research on the Tibetan Plateau and the impact of human activities has notably progressed^14,15^. Human activities and their ecological impacts on the plateau have been extensively investigated. In particular, with the support of RS and GIS technologies and related data, significant advancements have been achieved in research on land use change, ecological degradation, and regional ecological security^16,17^. However, despite these studies, research on the spatial differences in human activity space occupation and its ecological impacts on the Tibetan Plateau is relatively scarce. Domestically, the focus has primarily been on the ecological and environmental changes on the plateau, especially the impact of land use and land cover changes on the ecosystem. Studies have indicated that the ecosystem of the Tibetan Plateau is very sensitive to climate change and human activities. Agricultural expansion, urbanization, and other land use changes have significantly impacted the ecosystem. Nonetheless, contemporary studies exhibit a paucity of comprehensive examinations of the varying effects of human activities across diverse regions of the plateau.

The relationship between ecological changes on the Tibetan Plateau and human activities has received significant attention in recent studies. Several scholars, such as Guo^18^, Li^19^, and Zhang^20^, have analyzed the effects of climate change and human activities on ecosystems. Other researchers, such as Luo^21^, have highlighted the close relationship between climate factors, human activities, and net primary productivity (NPP), proposing a valuable enhanced CASA framework for estimating the NPP. Guo et al.^22^ used the centroid model and Geodetector to analyze the intertwined impacts on vegetation productivity but overlooked other potential factors and recent trends. Several scholars, such as Li^4^, have studied the impacts of climate change and human activities on desertification, summarizing four views on the dynamics of the alpine grasslands on the Tibetan Plateau^23^. Grassland ecosystems have also been considered; for instance, Wang^24^ and Xiong^25^ emphasized climate change as a major driver of grassland degradation and the key role of human activities in grassland restoration. However, they did not specify which human activities dominated nor did they provide specific ecological restoration measures. Similarly, Zhou^26^, Xiong^27^, and Wang^28^ studied the long-term trend of grassland ecosystems, emphasizing the positive role of human activities in grassland conservation. However, quantifying the contributions of various factors to grassland productivity remains a challenge. Liu^29^ used multiple methods to explore the spatiotemporal dynamics of the grassland NPP, but additional field validation may be needed to determine the factors affecting this process. Liu^30^ evaluated the effect of ecological conservation plans on the grassland NPP but did not examine the effectiveness of specific measures separately. Moreover, Liu^31^ focused on the grazing intensity and photosynthetically active radiation in relation to ecological changes. Sun^32^ comprehensively assessed the impact of human activities on alpine ecosystems, providing a new perspective on the relationship between human activities and ecosystems. However, whether all the data truly reflect the impact of human activities remains unclear^33^. In many studies, climate change, human activities, and ecological restoration strategies are considered key factors impacting the ecology of the Tibetan Plateau^34–40^. However, the space occupied by human activities, one of the most crucial factors, has not been systematically studied in terms of its ecological impact on the plateau. In this research, various forms of spatial occupation by different types of human activities were considered, focusing on construction land, arable land, and zonal vegetation for studying the ecological impact. Specifically, the ecological impact was characterized by calculating carbon sequestration and oxygen release, aiming to select and derive reliable data and assessment methods to comprehensively quantify the differing impacts of human activities on various ecosystems on the Tibetan Plateau.

**Figure 1:**
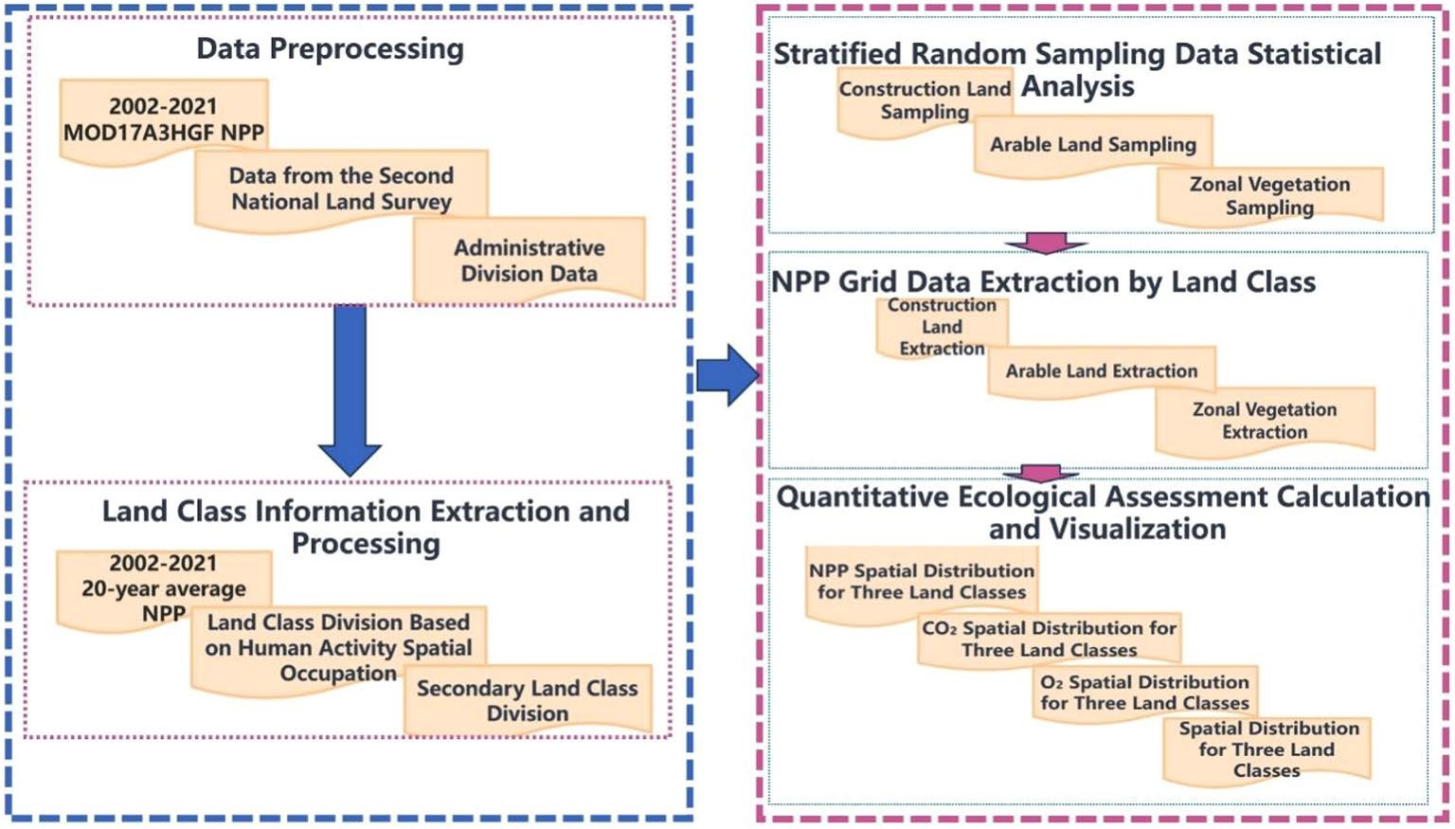
Workflow for analyzing the ecological impact of land type differences in human activity on the Tibetan Plateau.

Through quantitative assessment equations and spatial data, the differences in the ecological impacts of the various human land uses on the Tibetan Plateau from 2002 to 2021 were analyzed. With the utilization of land use data and multiyear average NPP data for the twenty-first century and by analyzing the spatiotemporal characteristics of construction and arable land on the Tibetan Plateau from 2002–2020, a quantitative assessment model for the ecological impact of construction land based on raster units was established, with empirical application research in Chengguan District, Lhasa. The workflow for analyzing the differences in land types concerning the ecological impact of human activities on the Tibetan Plateau is shown in Fig. 1. Through quantitative analysis and visualization of the NPP for the different land types on the Tibetan Plateau, the differences in each ecosystem were comprehensively evaluated, providing data and theoretical support for future ecological protection and sustainable development on the Tibetan Plateau.

## Research methods, data, and study area

### Research methods

In this study, the analysis of land use and land cover change (LUCC) was integrated with net primary productivity (NPP) data to assess the ecological impact of human activities on the Tibetan Plateau. Our methodology comprises two main components:

1. Land type difference analysis: To evaluate the ecological impact, we analyzed NPP data across different land use types on the Tibetan Plateau. This analysis was conducted using the following two approaches:
  ① Stratified random sampling: We employed stratified random sampling to select representative areas from different land use types for detailed analysis.
  ② Raster data extraction: With the use of raster data, we extracted NPP values corresponding to specific land use types to assess their ecological impacts.
2. Quantitative assessment model for the ecological impact: We developed a quantitative assessment model based on the photosynthetic reaction equation. This model was used to calculate the ecological impact of various land use types, providing a measure of the extent to which human activities affect the ecosystem of the Tibetan Plateau.

### Research data

In this study, we primarily used NPP data from the MOD17A3HGF v061 dataset developed by the NASA Data Center. This dataset provides the global terrestrial vegetation net primary productivity, validated against ground reference points, with a spatial resolution of 500 m and an annual temporal resolution. The dataset encompasses global land areas. Moreover, the data were collected by the Terra satellite and are provided in GeoTIFF format. The data originate from the MODIS sensor of NASA and are provided by the US Geological Survey (USGS). Land use data were sourced from the result database of the Second National Land Survey. The data preprocessing procedure included defining the projection as CGCS-2000, converting the data into the ESRI grid file format, and using Tibetan Plateau boundary data as a mask in the environmental settings. We analyzed NPP data from 2002 to 2021 to generate 20-year average data. We employed a series of spatial interpolation techniques to standardize the resolution of the different datasets, ensuring consistency across our various analyses. This involved the resampling of finer-resolution data to match the coarser-resolution datasets and applying geospatial alignment procedures to ensure accurate overlay and integration.

### Overview of the study area

**Figure 2:**
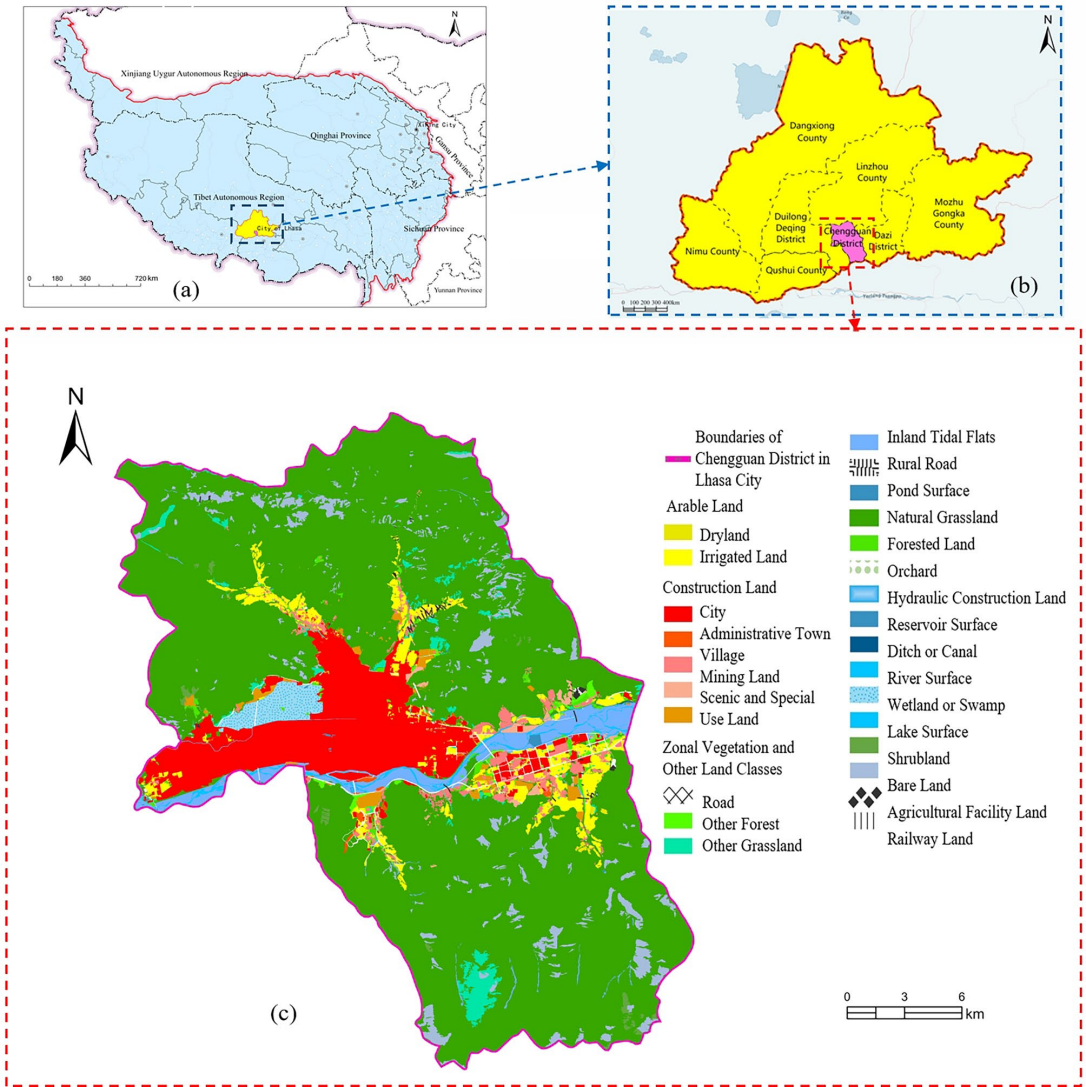
Overview of the research area. (**a**) Tibetan Plateau, (**b**) City of Lhasa, (**c**) Land use distribution map of Chengguan District, Lhasa city. Figure was created using ArcGIS Pro software (Version 3.0, Esri, Redlands, CA, [https://www.esri.com/en-us/home]). This map incorporates boundary data of the Tibetan Plateau, county-level administrative division data, and data from the Second National Land Survey of the Tibet Autonomous Region. The geographic representations, including dots and polygons for the administrative divisions within the Tibetan Plateau, were sourced from the National Catalogue Service for Geographic Information ([http://www.webmap.cn/commres.do?method=result100W]). We began by delineating the spatial extent of our study area. Subsequently, we overlaid the administrative division boundaries with the national land survey data. The resulting map was classified and color-coded according to various land use types and coverage conditions, thereby clearly depicting the spatial distribution of different land categories within the specified region.

Chengguan District is located in the central part of the Tibet Autonomous Region, with geographical coordinates of approximately 29° 39′ N and 91° 08′ E. An overview of the study area is shown in Fig. 2. The region predominantly exhibits plateau terrain features, with a high-to-low altitude gradient from south to north, demonstrating a stepped distribution and an average altitude of approximately 3650 m. Mostly sunny throughout the year, the area receives minimal rainfall, categorized as a plateau temperate semiarid climate. The annual highest temperature is 29.6 °C, and the lowest temperature is − 16.5 °C, with an annual average temperature of 8 °C and an annual precipitation of approximately 500 mm. Rainfall is concentrated in July, August, and September, with largely night rains. Chengguan District is rich in water resources and houses rivers such as the Lhasa River and Qushui River. The Lhasa River, a major water body in the area, flows through the city. Serving as the political, economic, cultural, and transportation hub of the Tibet Autonomous Region, Chengguan District fulfills a pivotal role. It encompasses 12 street offices and 50 village (residential) committees, with a total population of approximately 300,000 people. The economy is primarily driven by the tertiary sector, which is experiencing rapid development in the tourism and trade sectors. The primary and secondary industries are dominated by agriculture, pastoralism, and construction, respectively.

Chengguan District spans a total land area of 519.11 square kilometers. Notably, urban, village, industrial, and mining areas account for 73.01 square kilometers or 14.05% of the total land area, arable land accounts for 17.09 square kilometers or 3.29% of the total area, and zonal vegetation and other land types cover an area of 429.01 square kilometers, accounting for 82.66% of the total area. This district was selected as the study area due to its high representativeness. First, its unique geographical location in the heart of the Tibetan Plateau offers typical plateau terrain and ecological features. Second, the district is notable in terms of economic development, with a representative economic structure and industrial layout. Finally, Chengguan District features distinct regional characteristics of human activities and land use changes, revealing a clear zonal distribution pattern. The different land types exhibit distinct zonal spatial distributions, rendering them typical samples for studying human–land relationships on the Tibetan Plateau.

Chengguan District can be employed as a representative case for understanding the ecological dynamics of the Tibetan Plateau. While acknowledging that Chengguan District may not encompass the entire range of ecological conditions across the plateau, it provides a unique opportunity to study the impacts of urbanization and land use changes in a high-altitude urban setting. The insights gained from our focused study of Chengguan District can provide a critical understanding that can be used as a basis for broader comparative studies across the different regions of the Tibetan Plateau. By starting with a detailed case study, we can build a foundation to inform and guide future research endeavors aimed at explaining the diverse ecological impacts across the wider plateau region. This approach conforms with our goal of gaining meaningful and context-specific insights into the ecological impacts of land use changes, which can be instrumental in informing sustainable urban development strategies not only in Chengguan District but also in other similar high-altitude urban areas.

## Ecological impact quantitative assessment

### Theoretical background

The concept of net primary production (NPP) was initially proposed by ecologist Evelyn Hutchinson and has since become a pivotal idea in the field of ecology, serving as a descriptor of the productivity level of producers within an ecosystem and their capacity for carbon cycling. The NPP refers to the net primary production, also referred to as the net biomass production, representing the net carbon (C) fixed by plants in the photosynthesis process. Thus, the NPP represents the carbon (C) portion of organic compounds formed when plants harness light energy to combine carbon dioxide and water minus the carbon released during plant respiration. Consequently, the NPP is an indicator of the net carbon accumulation in plants within an ecosystem and is a vital metric when assessing ecosystem productivity and carbon cycling. The NPP of ecosystems exhibits spatiotemporal heterogeneity. In various regions and at different times, plant growth is influenced by a multitude of factors, such as climate, soil, precipitation, and sunlight. Hence, the NPP might significantly vary across different regions and time frames. For instance, the NPP of tropical rainforests vastly differs from that of grasslands. Similarly, at a single locale, seasonal climatic variations could cause changes in the NPP across different times. Moreover, the methods and precision of NPP measurements could differ depending on the research methodology employed.

### Quantitative assessment of carbon fixation and oxygen release

Photosynthesis is the process by which plants and certain other organisms convert carbon dioxide and water into organic compounds while releasing oxygen. Photosynthesis can be expressed as follows:

1 $$ 6CO_{2} + 6H_{2} O\mathop{\longrightarrow}\limits^{{light{\kern 1pt} {\kern 1pt} energy}}C_{6} H_{12} O_{6} + 6O_{2} . $$

In Eq. (1), carbon dioxide and water are transformed into glucose and oxygen under the influence of light. Photosynthesis comprises two main stages: light-dependent reactions (in which light energy is converted into transient chemical energy) and dark reactions (in which transient chemical energy is transformed into stable chemical energy).

The net primary production (NPP) represents the net amount of carbon (C) fixed by plants and other autotrophic organisms in the photosynthesis process. To compute the net glucose production, net carbon dioxide uptake, and net oxygen production, we employed the stoichiometry of the photosynthetic reaction. This approach involves leveraging the law of mass conservation in photosynthetic chemical reactions and using the relative atomic mass of each element.

Based on the law of mass conservation o and the relative atomic mass of each element, during photosynthesis, the proportion of carbon (C) in $C_{6} H_{12} O_{6}$ is 2/5, the proportion of carbon (C) in $CO_{2}$ is 3/11, and the proportion of oxygen (O) in $CO_{2}$ is 8/11. Given that the annual NPP is the mass of net C, based on the above proportions, the masses of $C_{6} H_{12} O_{6}$, $CO_{2}$ and $O_{2}$ can be calculated as follows:

2 $$ M(C_{6} H_{12} O_{6} ) = M(C) \times \frac{5}{2} = NPP \times \frac{5}{2}, $$

3 $$ M(CO_{2} ) = M(C) \times \frac{11}{3} = NPP \times \frac{11}{3}, $$

4 $$ M(O_{2} ) = M(CO_{2} ) \times \frac{{8}}{11}. $$

Substituting Eq. (3) into Eq. (4) yields the following result:

5 $$ M(O_{2} ) = M(C) \times \frac{11}{3} \times \frac{{8}}{11}{ = }NPP \times \frac{{8}}{3}. $$

## Data processing and result analysis for Chengguan district, Lhasa city

### Setting the sampling points and data collection via sampling point analysis

**Figure 3:**
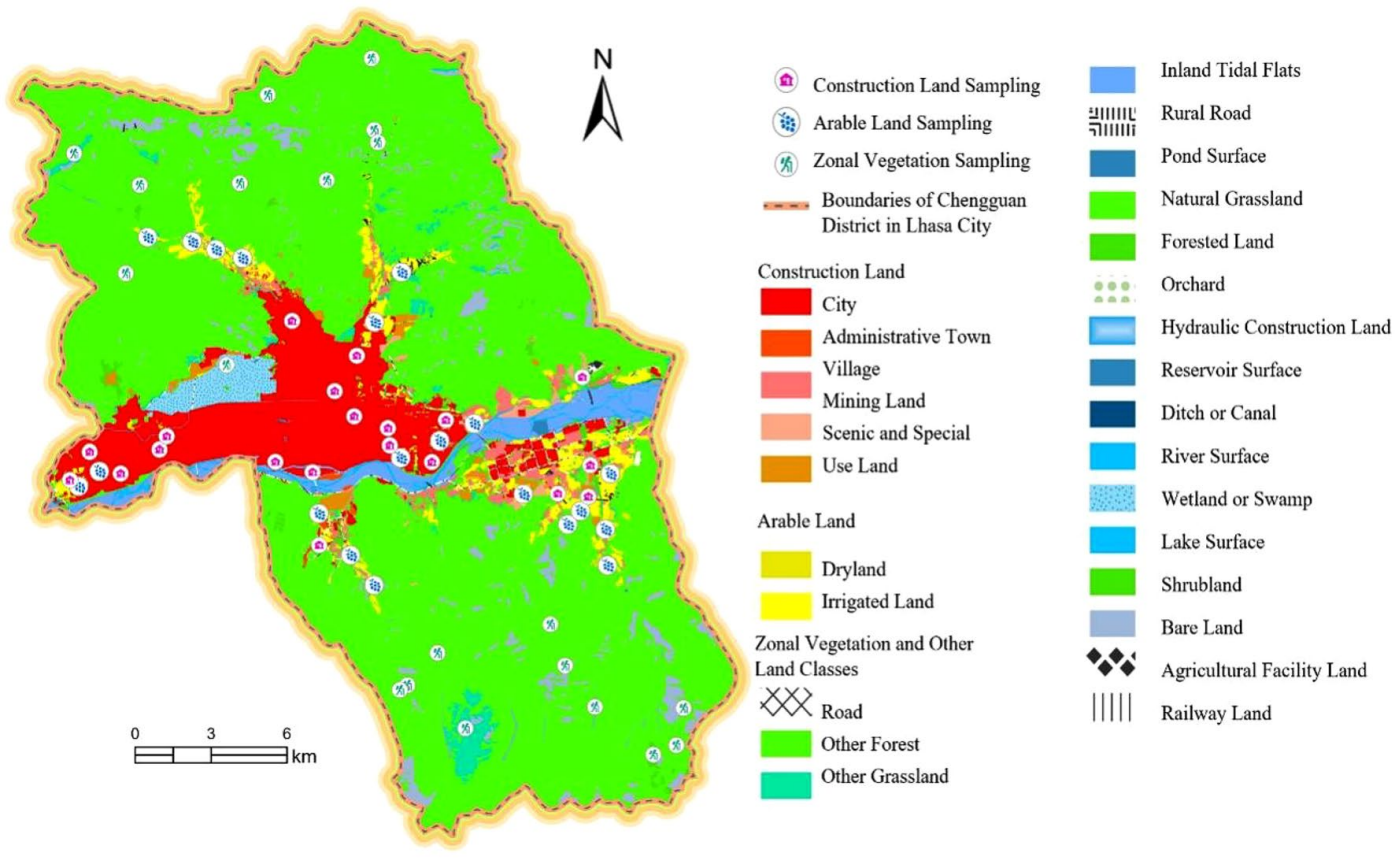
Distribution map of the secondary land types and sampling points in Chengguan District, Lhasa city. Figure presents the distribution map of land types and sampling points within Chengguan District, Lhasa City, created using ArcGIS Pro software (Version 3.0, Esri, Redlands, CA, [https://www.esri.com/en-us/home]). This map utilizes the same county-level administrative division data, and data from the Second National Land Survey of the Tibet Autonomous Region as Fig. 2. The visual representation of the administrative divisions within the Chengguan District is further enriched by the superimposition of sampling points, which were strategically placed across different land types to assess the land use dynamics. Each point and polygon on the map is color-coded and symbolized to correspond with the various secondary land types as categorized in the legend, providing a clear and detailed illustration of land use and vegetation cover in the area.

To quantitatively assess the regional differences in the spatial occupation and ecological impacts of construction and arable land in Chengguan District, Lhasa city, we selected 20 sampling points each from the zonal vegetation, construction land, and arable land categories, for a total of 60 points. The location of each sampling point is determined by the *x* and *y* coordinates, and the corresponding net primary productivity (NPP) data were recorded. The distribution of the sampling points is shown in Fig. 3 and summarized in Table 1.

**Table 1:** Sampling points in Chengguan district, Lhasa city.

Sampling point number (land type identified separately)	Land type	Secondary land type	NPP (kg/m^2^)
1	Arable land	Dry land	0.31
2	Arable land	Irrigated land	0.29
3	Arable land	Dry land	0.34
4	Arable land	Irrigated land	0.21
5	Arable land	Dry land	0.23
6	Arable land	Irrigated land	0.29
7	Arable land	Irrigated land	0.28
8	Arable land	Irrigated land	0.30
9	Arable land	Irrigated land	0.33
10	Arable land	Irrigated land	0.30
11	Arable land	Irrigated land	0.40
12	Arable land	Irrigated land	0.35
13	Arable land	Irrigated land	0.26
14	Arable land	Irrigated land	0.25
15	Arable land	Irrigated land	0.23
16	Arable land	Irrigated land	0.18
17	Arable land	Irrigated land	0.20
18	Arable land	Irrigated land	0.31
19	Arable land	Irrigated land	0.23
20	Arable land	Irrigated land	0.29
1	Construction land	City	0.23
2	Construction land	City	0.23
3	Construction land	City	0.17
4	Construction land	City	0.25
5	Construction land	City	0.10
6	Construction land	City	0.16
7	Construction land	City	0.20
8	Construction land	City	0.14
9	Construction land	City	0.13
10	Construction land	City	0.10
11	Construction land	City	0.16
12	Construction land	City	0.15
13	Construction land	City	0.23
14	Construction land	City	0.18
15	Construction land	Village	0.29
16	Construction land	Village	0.32
17	Construction land	Village	0.26
18	Construction land	Administrative town	0.27
19	Construction land	Mining land	0.16
20	Construction land	City	0.29
1	Grassland	Other grassland	0.13
2	Other land types	Bare land	0.18
3	Grassland	Natural grassland	0.33
4	Water areas and water conservancy facility land	Inland tidal flats	0.24
5	Forest land	Shrubland	0.21
6	Grassland	Natural grassland	0.16
7	Grassland	Natural grassland	0.19
8	Other land types	Wetland/swamp	0.43
9	Grassland	Other grassland	0.12
10	Grassland	Natural grassland	0.26
11	Other land types	Bare land	0.22
12	Grassland	Natural grassland	0.22
13	Grassland	Natural grassland	0.30
14	Grassland	Natural grassland	0.17
15	Grassland	Natural grassland	0.19
16	Grassland	Natural grassland	0.33
17	Forest land	Shrubland	0.31
18	Grassland	Natural grassland	0.31
19	Grassland	Natural grassland	0.28
20	Grassland	Natural grassland	0.29

### Sampling point data processing and statistical description

**Figure 4:**
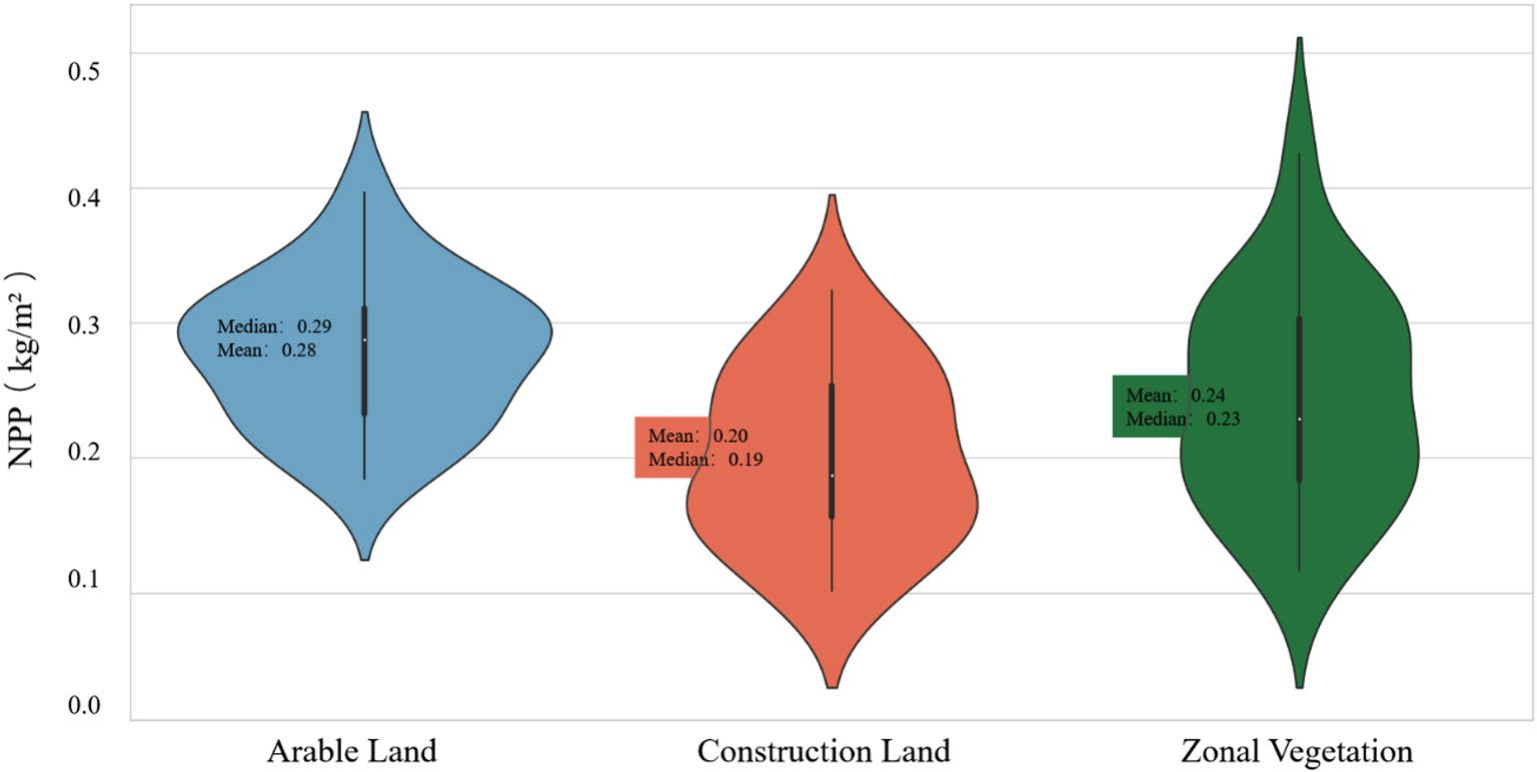
Violin chart of the NPP distributions at the different sampling points in Chengguan District, Lhasa city.

As shown in Fig. 4, we visualized the NPP data of the sampling points in the three land categories by utilizing violin plots to intuitively show their distribution characteristics and differences.

We rendered the NPP data of the sampling points provided in Table 1 for the three land categories separately into violin plots, aiming to intuitively showcase their distribution characteristics and differences. A violin plot is a data visualization tool that merges the features of box and density plots, serving to depict the data distribution along with the probability density. In this research, we employed violin plots to analyze the NPP distribution at the sampling points across the different land categories in Chengguan District, f Lhasa city. The symmetry of the violin plot allows us to effortlessly compare the NPP distribution across the various land categories. Such a comparison is especially meaningful in environmental and geographical research since it aids in identifying pronounced differences stemming from human intervention or natural changes.

Median: The white dot in the graph represents the median of the NPP data, offering a perspective on the central tendency of the data.

Quartiles: The black bars in the violin plot denote the upper and lower quartiles of the data, providing insight into the data distribution range.

Kernel density distribution: The width of the violin plot reflects the data density; a broader section indicates regions with a higher concentration of data points, while a narrower section indicates areas with fewer data points.

Outliers: If there are any outliers, they are usually depicted as separate dots outside the violin plot.

The generated plots can provide the following insights:

#### Kernel density estimation

The width of the violin plot reflects the kernel density of the NPP data for the different land types, indicating the distribution density of the NPP within various numerical ranges. A wider part of the violin represents a denser distribution of the NPP within the given value range, while a narrower section indicates a sparser distribution. By comparing the width variations of the violin plots for the different land types, we can analyze the differences in the NPP distribution density. For arable land, the sampling point kernel density is concentrated at approximately 0.3 kg/m^2^, with a dense distribution below 0.3 kg/m^2^ and a sparser distribution above 0.3 kg/m^2^. For construction land, the kernel density of the sampling points is concentrated at approximately 0.16 kg/m^2^, with a sparser distribution below 0.16 kg/m^2^. However, between 0.16 and 0.26 kg/m^2^, the distribution is more concentrated, and it becomes sparser above 0.26 kg/m^2^. The kernel density for the zonal vegetation sampling points is centered at approximately 0.19 kg/m^2^, with a dense distribution between 0.2 and 0.3 kg/m^2^, while the distributions below 0.19 kg/m^2^ and above 0.3 kg/m^2^ are sparser. The NPP value decreased overall with the transformation of zonal vegetation into construction land. In contrast, the NPP increased overall with the transformation of zonal vegetation into arable land. Arable land exhibits an increase in the lower NPP portions, but the originally higher NPP values of zonal vegetation are minimally affected by arable land.

#### Median and mean

Within each violin plot, the white dots represent the median NPP for this specific land type, with the mean also noted, visually reflecting the central tendency of the NPP across the different land types. By comparing the medians of the different land types, we can determine the differences in the NPP. The median NPP for arable land was 0.29 kg/m^2^, with a mean of 0.28 kg/m^2^. For construction land, the median NPP was 0.19 kg/m^2^, with a mean of 0.20 kg/m^2^. The median NPP for zonal vegetation was 0.23 kg/m^2^, with a mean of 0.24 kg/m^2^. The NPP of construction land notably decreased compared to that of zonal vegetation, while the NPP of arable land significantly increased relative to zonal vegetation.

#### Quartiles

The black lines in the violin plots are boxplots. The thicker parts of the black lines represent the interquartile range, revealing the dispersion degree of the NPP data. A larger interquartile range indicates greater data fluctuation, while a smaller interquartile range indicates less fluctuation. The thinner lines at the ends are whiskers, which extend to the maximum and minimum data values. By merging boxplot analysis and kernel density estimation, the violin plot allows us to simultaneously perceive the data distribution and density. The NPP data dispersion for natural land types is extensive, with interquartile ranges of 0.18 and 0.31 kg/m^2^. The dispersion degree of the NPP data for arable and construction land, both influenced by human activities, notably decreased. For construction land, the NPP values exhibited an interquartile range of 0.15 to 0.25 kg/m^2^. Transforming zonal vegetation into construction land resulted in a decrease in surface vegetation, causing a considerable reduction in the NPP. In the case of the arable land, the interquartile range is 0.24 to 0.31 kg/m^2^. The NPP values significantly increased after crop cultivation in zonal vegetation areas with lower NPP values. However, natural areas with originally higher NPP values, such as natural grasslands, did not experience a notable increase relative to crop areas. Notably, arable land exerted a pronounced positive effect on zonal vegetation areas with lower NPP values.

#### Variation range

The variation range of the NPP for zonal vegetation, i.e. 0.12 to 0.43 kg/m^2^, was notably greater than that for construction land (0.10 to 0.33 kg/m^2^) and arable land (0.18 to 0.40 kg/m^2^). These figures reflect the NPP value ranges for natural zonal vegetation, construction land, and arable land. The given ranges indicated the following: Variation Range: The variation range of the NPP values of natural zonal vegetation is the largest among the three land use types, indicating higher variability or diversity in the ecological productivity. Ecological Productivity: Natural zonal vegetation exhibited the highest maximum NPP value and a almost lowest minimum NPP value. The NPP value range of construction land was smaller, indicating that human activities caused a decline in ecological productivity in the region. Although agricultural activity enhanced the ecological productivity in certain areas, it still remained below the maximum values of the original vegetation. Ecological Impact: The wide NPP value range of natural zonal vegetation suggested that this region possesses superior ecological stability and adaptability. The smaller NPP value range of construction land indicated that human-induced disturbances reduced the ecological productivity in the region. The NPP values for arable land suggested that human activities increased the ecological productivity in certain areas, but this trend was not always positive, as an exceedingly high NPP could lead to imbalances in certain ecosystem functions. In summary, these data suggested that the original vegetation provided the most extensive range of NPP variation since it was relatively unaffected by extensive human intervention. Moreover, anthropogenic activities, such as construction and agriculture, yielded either positive or negative impacts on ecological productivity, depending on the specific activity and location.

### Quantitative calculation and visualization of carbon sequestration and oxygen release with different land type boundary extraction methods

NPP data were extracted based on the vector boundaries of the three land classes. Subsequently, Eqs. (2), (3), and (5) were used to calculate the masses of $C_{6} H_{12} O_{6}$, $CO_{2}$ and $O_{2}$, respectively, yielding raster data for the different land types.

**Figure 5:**
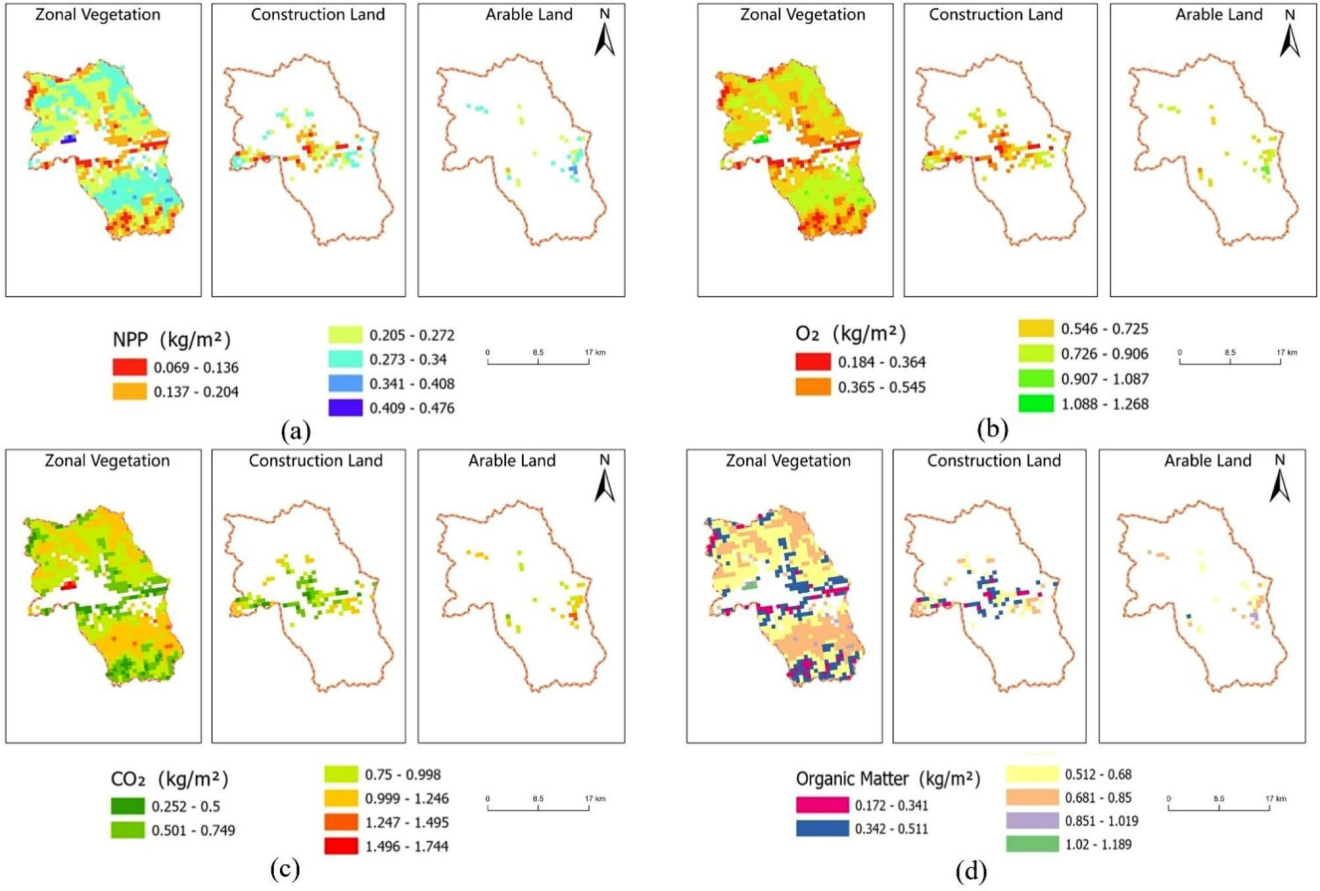
Grid distribution maps of the NPP, $CO_{2}$, $O_{2}$ and $C_{6} H_{12} O_{6}$ for the different land types. (**a**) NPP grid distribution map for the different land types (**b**) Oxygen grid distribution map for the different land types. (**c**) CO_2_ grid distribution map for the different land types (**d**) Organic matter grid distribution map for the different land types. Figure showcases grid distribution maps of Net Primary Productivity (NPP), CO_2_, O_2_, and organic matter across various land types within Chengguan District, Lhasa City. These maps were generated by conducting raster calculations in ArcGIS Pro software (Version 3.0, Esri, Redlands, CA, [https://www.esri.com/en-us/home]). The base NPP data utilized for these calculations were sourced from the MOD17A3HGF v061 dataset, which is available through the NASA Earth Data Center ([https://earthdata.nasa.gov/]). Boundary data were acquired from the National Catalogue Service for Geographic Information ([http://www.webmap.cn/commres.do?method=result100W]), and the land class boundaries were derived based on the second National Land Survey of the Tibet Autonomous Region. The quantitative assessment of carbon fixation and oxygen release, elaborated in Sect. “Quantitative assessment of carbon fixation and oxygen release” of the main text, forms the basis of the calculations for these distribution maps.

In this study, the raster distribution of the NPP for the different land classes, as well as the calculated carbon sequestration and oxygen release, enabled in-depth analysis of the ecological functions of each land type. The thematic raster distribution maps depicted in Fig. 5 elucidate the spatial distribution variations in the net primary productivity (NPP), carbon dioxide, oxygen, and organic matter among the different land types, as well as their spatial variation trends within each land type. By analyzing these maps and charts, the following conclusions can be obtained:

- Comparison of the NPP, carbon dioxide, oxygen, and organic matter distributions across the different land types. The ecological functional characteristics of each land type were determined by comparing their distributions. Notable differences in carbon sequestration and oxygen release were observed between construction land, arable land, and zonal vegetation. Human intervention reduced the randomness of the data and decreased the range of the carbon sequestration and oxygen release values. Notably, there was a pronounced difference in the carbon sequestration and oxygen release patterns between construction and arable land relative to zonal vegetation. Human activities, especially farming and construction, changed the NPP and carbon sequestration/oxygen release functions in these areas. This could be attributed to land transformation and land use changes, which affects the vegetation structure, biodiversity, and ecological functions.
- Intra-land type ecological system changes and analysis of the driving factors. On the Tibetan Plateau, the ecological functions within each land type exhibited specific spatial distribution trends. These trends were not mere random distributions. They resulted from a combination of natural and anthropogenic factors. For example, the NPP distribution in construction land may be linked to the land use type, population density, and infrastructure distribution. However, in arable land, the NPP variations might be related to the land use type, specific crop patterns, land management practices, and crop rotation systems. An in-depth examination and correlation analysis of these distributions could provide a robust basis for formulating land use and ecological protection policies.
- Zonal influence and the role of geographical environmental factors in ecological system functions. On the Tibetan Plateau, geographical environmental factors such as climate, soil, and terrain significantly influence the distributions of the NPP, carbon dioxide, oxygen, and organic matter. In particular, when zonal vegetation is combined with other vegetation types, it provides a complex picture of its ecological system function. In future research, it would be vital to further integrate climate models, soil databases, and remote sensing data to provide a comprehensive analysis at finer spatial and temporal scales. This study revealed the intricacies of the ecological system functions on the Tibetan Plateau and their interrelationships with geographical environmental factors.

### Analysis of the NPP in subcategories under the different land classes

Initially, we conducted data preprocessing, encompassing tasks such as data cleaning and anomaly detection. Subsequently, we calculated descriptive statistical measures, including the mean, maximum, and minimum values of the NPP under each land class, to comprehensively obtain the fundamental distribution characteristics of the NPP across the various land classes. Furthermore, we segmented and statistically analyzed the subcategories of the three main land classes and calculated the maximum, minimum, and average NPP values for each subcategory. The results are provided in Table 2. Through detailed statistical analysis of the NPP data in the above subcategories, we investigated the differences in ecosystem functions under the different land classes.

**Table 2:** Statistical data of the NPP of the secondary land classes.

Secondary land class name	Minimum	Maximum	Average
Shrubland	0.23	0.23	0.23
River surface	0.07	0.27	0.13
Lake surface	0.07	0.07	0.07
Pond surface	0.18	0.18	0.18
Bare land	0.11	0.34	0.24
Inland tidal flats	0.07	0.31	0.14
Other grassland	0.09	0.26	0.18
Other grassland	0.10	0.21	0.15
Natural grassland	0.10	0.36	0.25
Forest land	0.11	0.30	0.22
Wetland/swamp	0.34	0.48	0.42
Mining land	0.09	0.23	0.15
City	0.07	0.33	0.20
Village	0.14	0.32	0.25
Scenic and special use land	0.14	0.33	0.25
Administrative town	0.09	0.23	0.16
Irrigated land	0.20	0.40	0.28

In our analysis of the data, we examined the ecological feature variations across the different subcategories from several distinct perspectives, including ecological function, land use impact, and spatial distribution. This comprehensive approach allowed us to better understand the interplay between human activities, land use changes, and their ecological consequences. By comparing the average NPP of each subcategory, we identified marshlands, irrigated lands, and natural grasslands as exhibiting higher productivity, while lakes, river surfaces, and inland shoals exhibited lower productivity. Notably, the extremely low and high values of the NPP in the zonal vegetation area are more pronounced than those in the other land classes affected by human activities, indicating the significant impact of human activities on plateau ecosystems.

**Figure 6:**
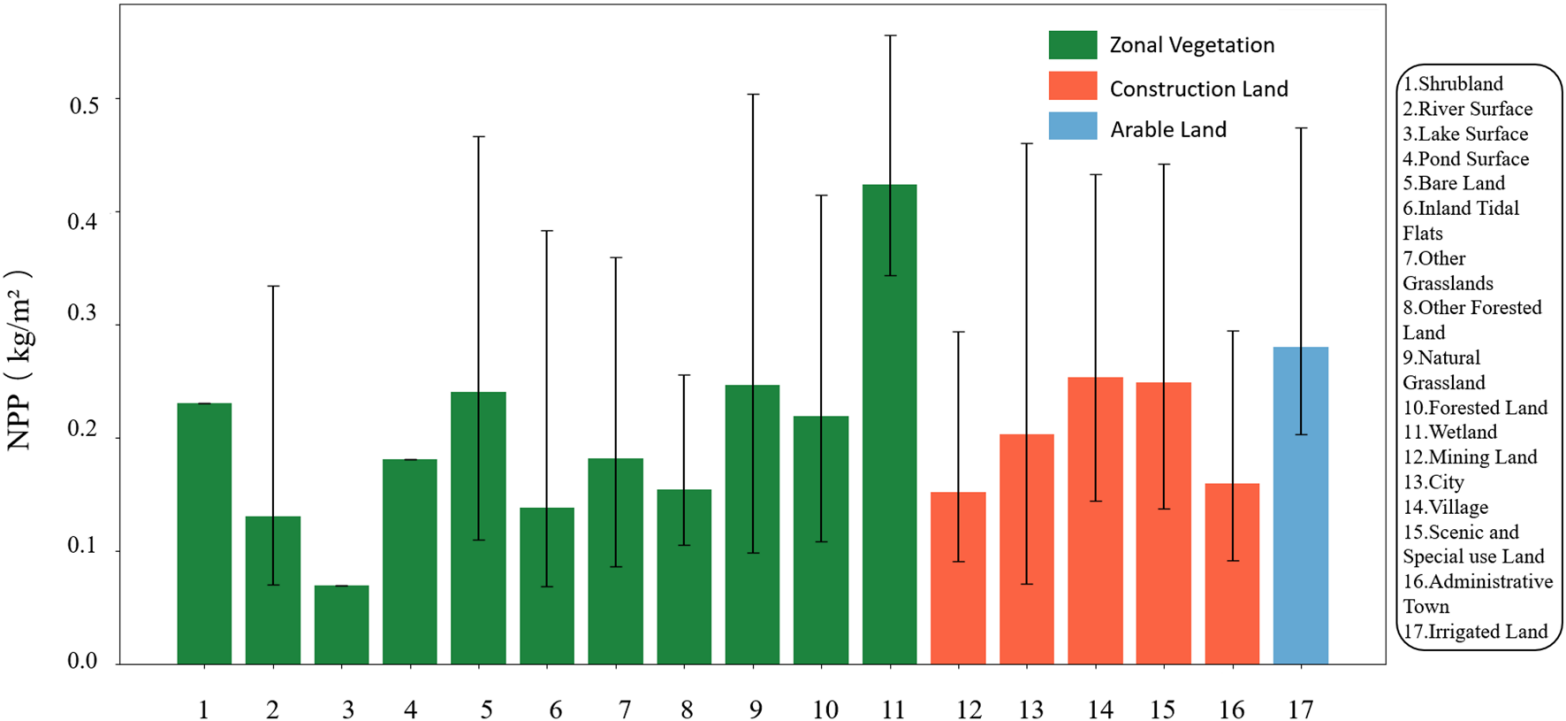
Average NPP and maximum and minimum NPP values for the different land types. The amount of dry land data is too small to generate effective raster data, resulting in a lack of dry land data.

To visualize the statistical outcomes of the NPP under the subcategories of land use classification, we generated bar charts with error bars (Fig. 6). The chart displays the maximum, minimum, and average values of the NPP for the different subcategories, as well as the range of NPP fluctuations, reflecting the amplitude of NPP variation in each land class. The error bar charts offer a more intuitive portrayal of ecosystem variations across the different subcategories, providing a greater understanding of the impact of human activities on the various ecosystems.

Figure 6 shows that the results are largely consistent with those obtained using the sampling point extraction method. The NPP variability in land classes such as natural grasslands, urban areas, and bare lands was considerable, as evidenced by the larger lengths of the error bars. This finding suggested substantial differences in the NPP within these classes, likely influenced by various ecological conditions and human activities. Moreover, the subcategories with shorter error bars, such as shrublands, lake surfaces, and other forests, indicated a smaller range of NPP variability, suggesting stable NPP values in these categories, minimal fluctuation, or overlapping data points due to smaller sample sizes.

Furthermore, by comparing the error bars across the different land classes, we could determine the differences in the range of NPP variability. For instance, the NPP variability in the zonal vegetation area was notably greater than that in the construction and arable land areas. This may occur because zonal vegetation comprises a wider variety of ecosystem types and hence is more susceptible to various uncertain factors. By analyzing Fig. 6, we identified the fluctuating characteristics of the NPP across the various subcategories and their relationships with the ecological conditions and human activities, which could support further research on the ecological impacts of regional differences on the Tibetan Plateau.

## Comprehensive assessment and discussion

In our study of Chengguan District, Lhasa city, we focused on examining the NPP variations across different ecosystems by employing both quantitative and visual analysis methods. Our key findings demonstrated that construction land in Chengguan District is primarily distributed along river valleys, with arable land predominantly surrounding this land use type, impacting the distribution of the NPP. The NPP of the original zonal vegetation ranged from 0.2 to 0.3 kg/m^2^. In contrast, the transformation into construction land caused a decrease in the NPP, with values ranging from 0.16 to 0.26 kg/m^2^. Moreover, arable land demonstrated an increase in the NPP, with average values above 0.3 kg/m^2^, suggesting enhanced productivity, especially in areas where the zonal vegetation initially exhibited lower NPP values. However, it is important to recognize that this apparent increase in productivity in arable land areas may not uniformly represent a positive ecological change. Such increases could potentially disrupt the natural balance of ecosystems, leading to unforeseen ecological consequences. For instance, while an increased NPP might indicate intensified agricultural practices contributing to short-term productivity increases, it may also raise concerns regarding long-term sustainability and potential ecological imbalances, such as biodiversity loss or soil degradation. Furthermore, the wider range of the NPP values in the original zonal vegetation area than in the transformed vegetation area reflects the inherent resilience of natural ecosystems. This resilience enables these ecosystems to sustain diverse ecological functions under varying environmental conditions, a capability that might be diminished in areas affected by human activities. The uniformity of the NPP in the original zonal vegetation area and its disruption in human-modified areas highlight the complexity of the ecological impacts stemming from land use changes. This approach challenges the conventional narrative that associates increased productivity with positive ecological outcomes and highlights the need for a more holistic approach to understanding and managing these impacts.

This observed pattern of NPP variation, particularly the significant increase in arable land, may have broader ecological implications. While the higher variability in the NPP in the zonal vegetation area (ranging from 0.12 to 0.43 kg/m^2^) reflects its ecological stability and adaptability, the low variability in human-impacted arable and construction land areas indicates a potential change in ecosystem resilience. Our results, which differ from those of other studies reporting various impacts of land use changes on ecosystems in similar high-altitude regions, suggest that while human activities stabilize the ecosystem productivity, they might also reduce ecosystem resilience^41,42^. These insights emphasize the need for further exploration into the ecological impacts of human activities on the Tibetan Plateau.

While our study provides valuable insights into the NPP variations across the different ecosystems in Chengguan District, Lhasa city, it is essential to acknowledge certain limitations and uncertainties. The data used, primarily MODIS-derived NPP data and land use classification data, carry inherent limitations in the spatial resolution and classification accuracy. These factors could influence the granularity and precision of our findings. Additionally, our methodological approach, which focuses solely on spatial analysis, may not fully account for temporal variations and the complex ecological processes influencing the NPP.

Future research should reveal the complex interplay between ecological dynamics and anthropogenic influences on the Tibetan Plateau, particularly by focusing on how the increased net primary productivity (NPP) in arable land areas and its reduction in construction land areas impact long-term ecological processes. This exploration could be extended to include the effects on biodiversity, soil health, and overall ecosystem functionality. We aimed to analyze the influences of geographical and environmental factors, such as climate, soil, and topography, on various ecological indicators, including the NPP, carbon dioxide, oxygen, and organic matter distribution^43^. Our approach particularly focused on zonal vegetation, aiming to provide a nuanced understanding of the causes^44^ of ecosystem variations across different land classes. By integrating the study of natural environmental factors with human activities, our research strives to offer targeted recommendations for ecological conservation and sustainable development on the Tibetan Plateau and other ecologically sensitive areas worldwide.

## Data Availability

The data are available from the corresponding author on reasonable request.
