# Author Correction: Assessing the spatial occupation and ecological impact of human activities in Chengguan district, Lhasa city, Tibetan Plateau

**DOI:** 10.1038/s41598-024-58851-9

**Published:** 2024-04-10

**Authors:** Lin Xu, Yong Xu, Jian Duan, Yingying Wang, Hua Yang

**Affiliations:** 1 Key Laboratory of Regional Sustainable Development Modeling, Institute of Geographic Sciences and Natural Resources Research (IGSNRR), Chinese Academy of Sciences (CAS), Beijing, 100101 China; 2 College of Resources and Environment, University of Chinese Academy of Sciences, Beijing, 100049 China; 3 College of Geography and Environmental Sciences, Zhejiang Normal University, Jinhua, 321004 China; 4 Key Laboratory for Geographical Process Analysis and Simulation of Hubei Province and School of Urban and Environmental Sciences, Central China Normal University, Wuhan, 430079 China

Correction to: *Scientific Reports* 10.1038/s41598-024-57221-9, published online 23 March 2024

The original PDF version of this Article contained an error in Figure 2a, where a letter ‘C’ was incorrectly added to the panel.

The original Figure 2 and accompanying legend appear below.

**Figure 2:**
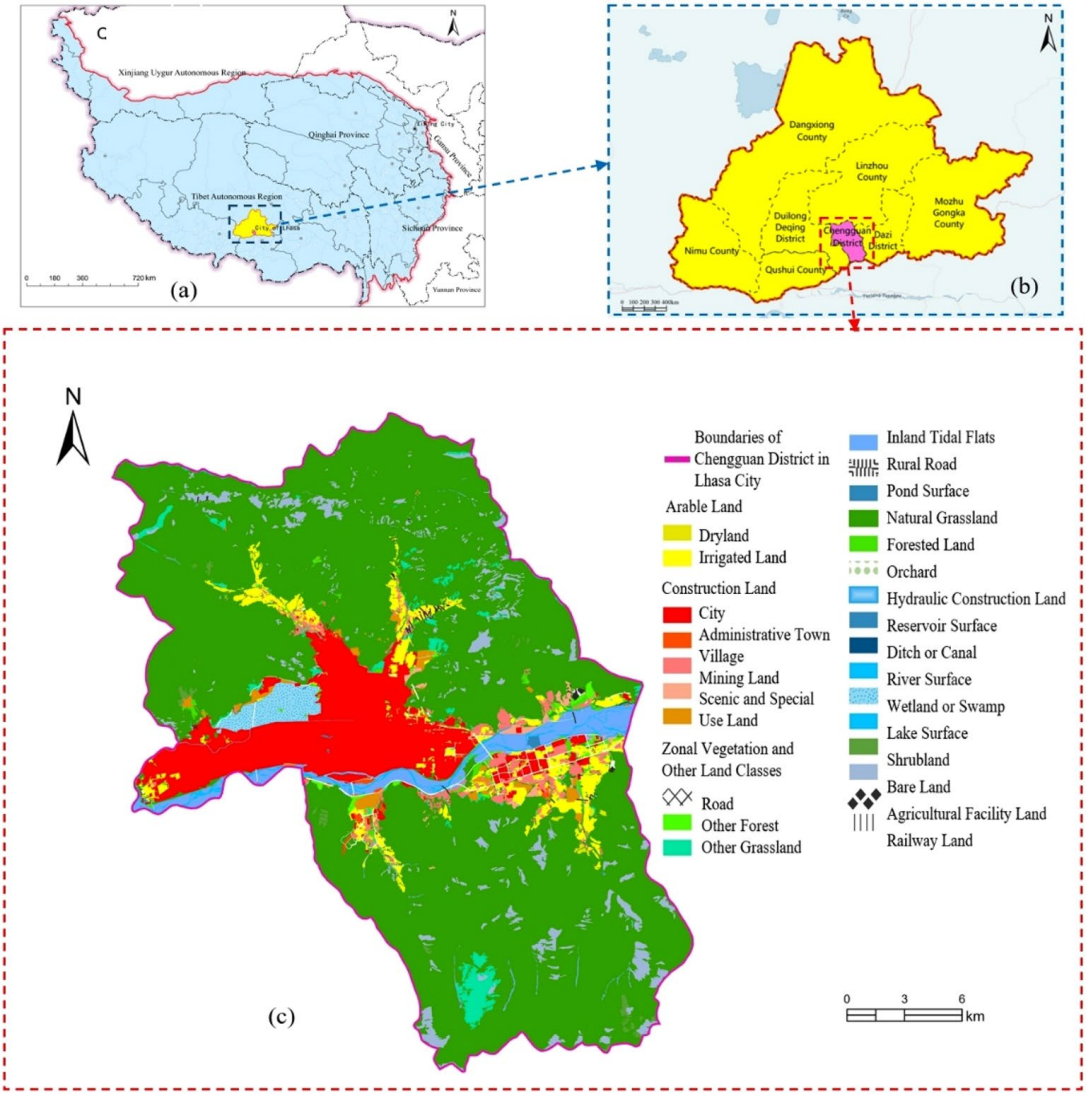
Overview of the research area. (**a**) Tibetan Plateau, (**b**) City of Lhasa, (**c**) Land use distribution map of Chengguan District, Lhasa city. Figure was created using ArcGIS Pro software (Version 3.0, Esri, Redlands, CA, [https://www.esri.com/en-us/home]). This map incorporates boundary data of the Tibetan Plateau, county-level administrative division data, and data from the Second National Land Survey of the Tibet Autonomous Region. The geographic representations, including dots and polygons for the administrative divisions within the Tibetan Plateau, were sourced from the National Catalogue Service for Geographic Information ([http://www.webmap.cn/commres.do?method=result100W]). We began by delineating the spatial extent of our study area. Subsequently, we overlaid the administrative division boundaries with the national land survey data. The resulting map was classified and color-coded according to various land use types and coverage conditions, thereby clearly depicting the spatial distribution of different land categories within the specified region.

The original Article has been corrected.

